# Profiling Chromatin Accessibility Responses in Goat Bronchial Epithelial Cells Infected with *Pasteurella multocida*

**DOI:** 10.3390/ijms24021312

**Published:** 2023-01-09

**Authors:** Qiaoling Chen, Zhen Chen, Zhenxing Zhang, Haoju Pan, Hong Li, Xubo Li, Qi An, Yiwen Cheng, Si Chen, Churiga Man, Li Du, Fengyang Wang

**Affiliations:** Hainan Key Laboratory of Tropical Animal Reproduction & Breeding and Epidemic Disease Research, Engineering Key Laboratory of Haikou, School of Animal Science and Technology, Hainan University, Haikou 570228, China

**Keywords:** ATAC-seq, chromatin accessibility responses, goat bronchial epithelial cell, *Pasteurella multocida*, immune reaction

## Abstract

*Pasteurella multocida* can cause goat hemorrhagic sepsis and endemic pneumonia. Respiratory epithelial cells are the first line of defense in the lungs during *P. multocida* infection. These cells act as a mechanical barrier and activate immune response to protect against invading pathogenic microorganisms. Upon infection, *P. multocida* adheres to the cells and causes changes in cell morphology and transcriptome. ATAC-seq was conducted to determine the changes in the chromatin open region of *P. multocida*-infected goat bronchial epithelial cells based on transcriptional regulation. A total of 13,079 and 28,722 peaks were identified in the control (CK) and treatment (T) groups (P. multocida infection group), respectively. The peaks significantly increased after *P. multocida* infection. The specific peaks for the CK and T groups were annotated to 545 and 6632 genes, respectively. KEGG pathway enrichment analysis revealed that the specific peak-related genes in the T group were enriched in immune reaction-related pathways, such as Fc gamma R-mediated phagocytosis, MAPK signaling pathway, bacterial invasion of epithelial cells, endocytosis, and autophagy pathways. Other cellular component pathways were also enriched, including the regulation of actin cytoskeleton, adherent junction, tight junction, and focal adhesion. The differential peaks between the two groups were subsequently analyzed. Compared to those in the CK group, 863 and 11 peaks were upregulated and downregulated, respectively, after the *P. multocida* infection. Fifty-six known transcription factor motifs were revealed in upregulated peaks in the *P. multocida*-infected group. By integrating ATAC-seq and RNA-seq, some candidate genes (SETBP1, RASGEF1B, CREB5, IRF5, TNF, CD70) that might be involved in the goat bronchial epithelial cell immune reaction to *P. multocida* infection were identified. Overall, *P. multocida* infection changed the structure of the cell and caused chromatin open regions to be upregulated. In addition, *P. multocida* infection actively mobilized the host immune response with the inflammatory phenotype. The findings provide valuable information for understanding the regulatory mechanisms of *P. multocida*-infected goat bronchial epithelial cells.

## 1. Introduction

*Pasteurella multocida* is one of the most prevalent commensal and opportunistic pathogens in domestic and wild animals globally [1]. Humans can be infected with *P. multocida* via animal bites or by handling respiratory secretions from diseased animals. Accordingly, *P. multocida* has an economic impact on the breeding industry and poses a threat to global public health and biosecurity [2]. The economically important diseases caused by this bacterium mainly include avian fowl cholera, rabbit snuffles, bovine hemorrhagic septicemia, and swine atrophic rhinitis, which are always caused by *P. multocida* and *Bordetella bronchiseptica* [3,4]. In sheep and goat, *P. multocida* can cause hemorrhagic septicemia and endemic pneumonia [4].

The pathogenic mechanisms of *P. multocida* can invade the mucosa, evade innate immunity, and cause systemic disease of the host [1,4]. *P. multocida* is always found to be resided in the oral, nasal, and respiratory cavities of animals [1,2]. The respiratory epithelium acts as the first line of defense against the invasion and stimuli of respiratory pathogens [5,6]. Subsequently, epithelial cells detect diverse pathogens through an ample repertoire of membrane-bound, endosomal and cytosolic pattern recognition receptors (PRR). Among them, PRR activation by pathogen-associated molecular patterns (PAMPs) or danger−associated molecular patterns (DAMPs) initiates a signaling cascade immune reaction of the host that can promote pathogen exclusion or expulsion, recruit and activate leukocyte−mediated defenses, directly kill microbes, and restore host homeostasis [7]. During infection from *P. multocida*, the host immune response reaction was also activated and host epithelial barriers dysfunction and inflammation was induced [8].

Immune signal activation and gene transcriptome changes in epithelial cells are responses to *P. multocida* infection [9,10]. The change in chromatin structure in the nucleus is one of the reasons for the differences in gene transcription [11]. The open chromatin region often allows regulatory elements, such as transcription factor, which bind to the promoter region to initiate the transcriptional expression of the gene [11]. Therefore, genome-wide open chromatin regions must be accessed in a particular physiological state, and transcription factors that cause changes in the host mRNA expression profile under the specific situation must be identified.

The Assay for Transposase−Accessible Chromatin with high−throughput sequencing (ATAC−seq) is a technique used in epigenetics and involves the cleavage of open nuclear chromatin regions in a specific space and time by transposase, which ultimately enables the acquisition of the active transcriptional regulatory sequences in the genome [12]. To date, limited studies have employed the ATAC−seq technology to explore changes in host cells infected with *P. multocida*. Therefore, in this study, ATAC−seq was employed to detect the open chromatin regions in *P. multocida*-infected goat bronchial epithelial cells compared to control cells, to identify the transcription factors that regulate the host immune reaction to pathogen invasion.

## 2. Results

### 2.1. Changes in Cell Morphology

**Figure 1:**
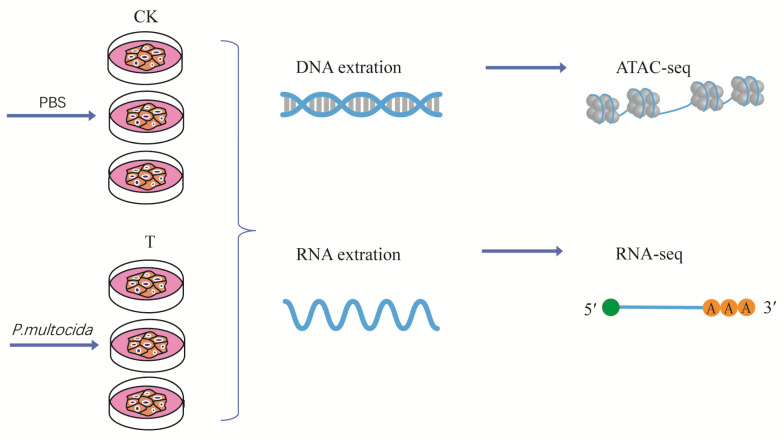
Overview of the experimental workflow. Three replicates of goat bronchial epithelial cells infected with *P. multocida* and three control replicates were collected for ATAC−seq. The same set of replicates was collected for RNA-seq.

**Figure 2:**
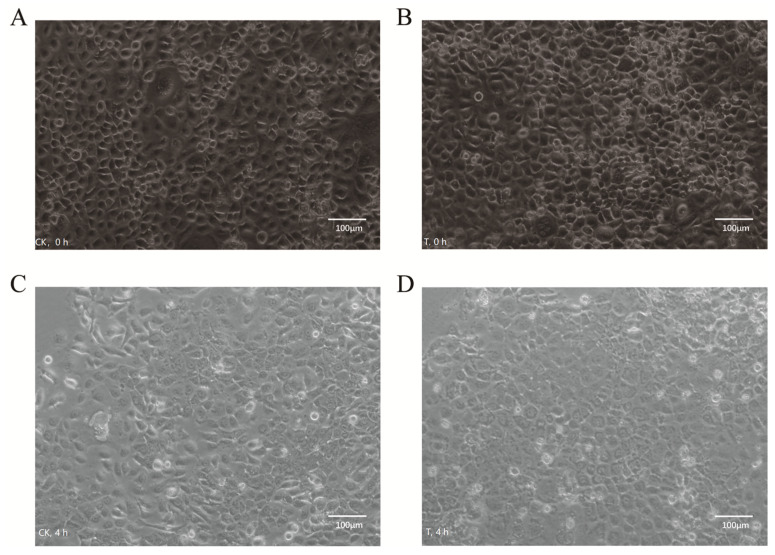
Image analysis of the cellular changes in goat bronchial epithelial cells incubated with *P. multocida* for 4 h. (**A**,**B**) Morphology of cells in the CK (Panel **A**) and T (Panel **B**) group at 0 h. (**C**,**D**) Morphology of cells in the CK (Panel **C**) and *P. multocida*-infected (Panel **D**) group at 4 h.

After 4 or 6 h of infection, three replicates of the 4 h treatment were digested and collected for nuclear extraction, and another three replicates treated for 6 h were used for RNA extraction (Figure 1). Goat bronchial epithelial cells were damaged after infection. In some areas, the cells appeared larger; the edges of cell membranes were blurred, roughened, or cracked, and diffuse fragments were recognized around the edge cells. Vacuoles were also commonly observed in cells. The cell morphology in the denser area of cell distribution was relatively uniform, and a small number of cell membranes appeared brighter. After 4 h of *P. multocida* infection, many dead cells were floating, some cells lost their regular paving stone shape, more dispersed fragments were found, and some cells were ruptured. Furthermore, cell membranes appeared rough. In contrast, control cells generally had a regular morphology, with smooth and complete cell membrane lines, and no obvious diffuse fragments (Figure 2).

### 2.2. ATAC−Seq Quality Control of the Goat Bronchial Epithelial Cells

**Figure 3:**
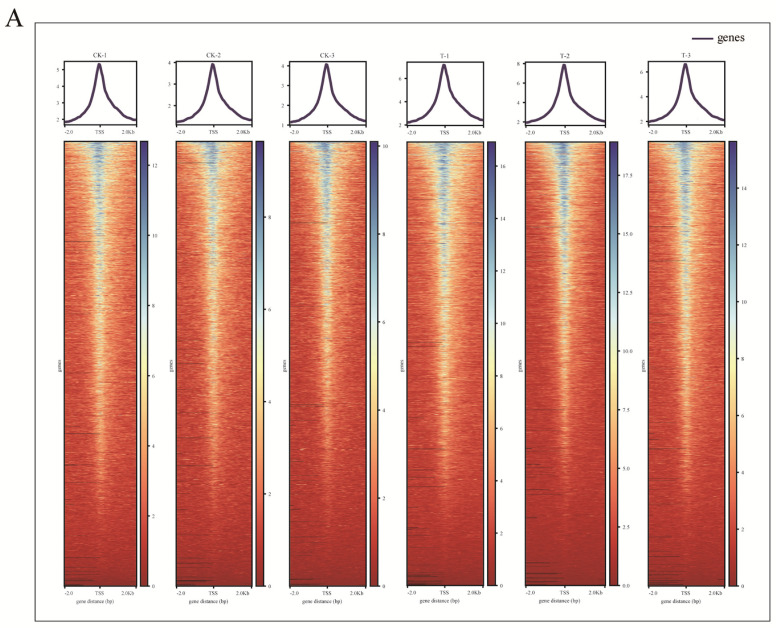

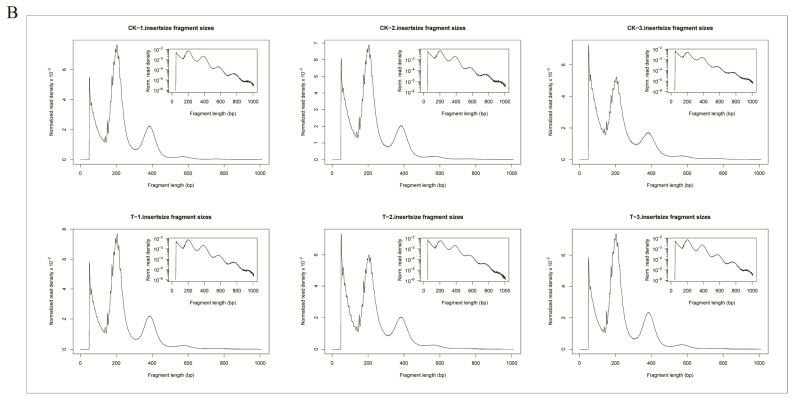
ATAC-seq quality control. (**A**) The top and bottom figures show the average read depth and enrichment of the ATAC−seq signals around the transcription start site (TSS), respectively. (**B**) Distribution of the fragment of each sample. The x axis represented the length of fragment, and the y axis represented the proportion of the fragment to all the fragments. The left−top was a nucleosome-free fragment corresponding to the open chromatin region (the nucleosome cleaved fragment < 147), and the highest peak on the right corner was the open chromatin fragment representing the fragment including two nucleosomes (the nucleosome cleaved fragment > 147 and <147 × 2) and another fragment including several nucleosomes, which is the characteristics of the open region of chromatin.

To determine the mechanism of infection of goat bronchial epithelial cells, ATAC−seq was performed to examine the accessibility of the genome. In this study, as shown in Table 1, 28,669,772−48,363,990 of raw reads for each sample were obtained. The ATAC-seq data information of each library before and after filtering is shown in Table 1. More than 84.73% goat genome mapping rates were obtained for the reads that mapped unique positions for downstream analysis. As shown in Figure 3A, statistical analysis of all reads in the 2 kb upstream and downstream of the TSS showed that the highest density was observed at the TSS, suggesting that the reads were distributed according to the typical characteristics of higher eukaryotes, and the open regions of chromatin were involved in transcriptional regulation. All libraries produced fragment lengths with the expected distribution and the insert fragment size analysis indicates the distribution of nucleosomes on chromatins (Figure 3B). The left-most was a nucleosome-free fragment corresponding to the open chromatin region (the nucleosome cleaved fragment < 147), and the highest peak on the right corner was the open chromatin fragment representing the fragment including two nucleosomes (the nucleosome cleaved fragment > 147 and <147 × 2) and another fragment including several nucleosomes, which is the characteristics of the open region of chromatin. Principal component analysis (PCA) also revealed the similarity between biological replicates and the difference between the two experimental groups (Figure S1).

### 2.3. Identification of Chromatin Open Regions by ATAC-Seq

**Figure 4:**
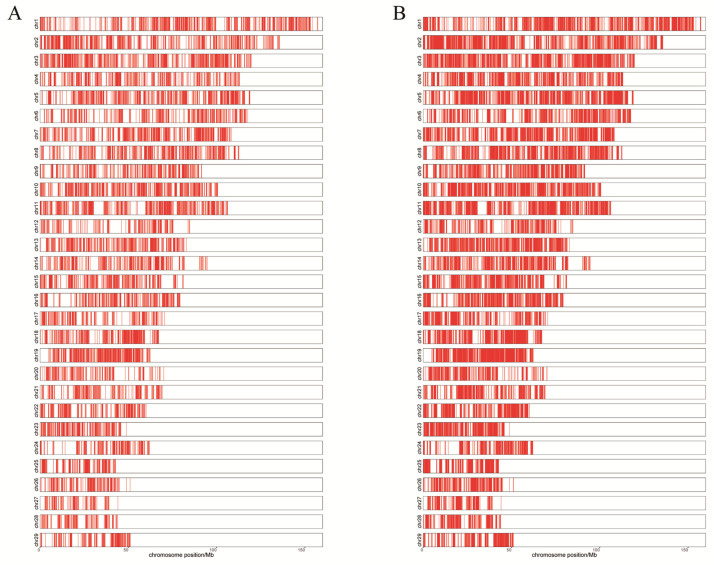

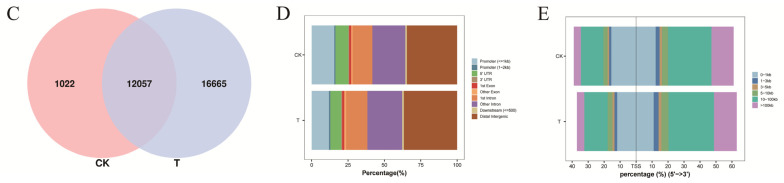
Analysis of the peaks in the CK and T groups. (**A**,**B**) Distribution of all peaks in chromosomes location landscape in the CK and T groups, respectively. (**C**) Venn diagram showing the overlapping peaks between the CK and T groups. (**D**) Location distribution of common peaks on the different regions of genome (promoter, 5′UTR, 3′UTR, exon, intron, downstream and distal intergenic) in CK and T group. (**E**) Common peaks relative TSS distance distribution ratio map in CK and T group.

Peaks were screened and identified for peak function analysis. As shown in Table 2, peaks were acquired for each sample. To further verify the most representative accessible regions of the genome among samples, the peaks that were at least commonly shared among two samples in same group were merged. As depicted in the distribution map of all peaks on the chromosome, the signal intensity of peaks in the treatment group was higher than that of peaks in CK group (Figure 4A,B). A total of 13,079 and 28,722 peaks were identified in the CK and T groups, respectively, with 12,057 common peaks (Figure 4C and Table 2). The peaks were annotated to the corresponding region of the genome. Notably, a similar pattern was observed for the peak distribution sites in the genome, with approximately one-third of peaks located in the distal intergenic of the genome (Figure 4D). Figure 4E shows that most of the peaks were concentrated at 10−100 kb from the TSS. In summary, the ATAC−seq data indicate that *P. multocida* infection changed the structure of the cell and caused the upregulation of chromatin open regions.

### 2.4. Peak−Related Gene Analysis and Annotation in Each Group

**Figure 5:**
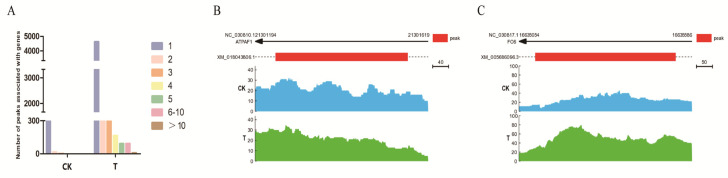
Analysis of specific peak-related genes in the CK and T groups. (**A**) Statistics for the peak numbers of genes in the different groups. (**B**) Visualization of the CK group-specific peak-related gene, ATPAF1. (**C**) Visualization of the T group-specific peak-related gene, FOS.

To explore the function of the peaks, those located less than 2 kb upstream from the TSS and no more than 500 bp downstream of TSS were analyzed using functional enrichment analysis, which considered them as promoter regions, playing important roles in regulating the genes’ expression. The specific peaks of the CK group were annotated to 545 genes, while those of the T group were annotated to 6632 genes. There were 304 overlapped genes between the two groups. Each gene had a different number of peaks. The peak number of each gene is shown in Figure 5A. In the CK group, only one gene had three peaks, 15 genes had two peaks and the remaining 529 genes had one peak. Among the 6632 genes in the T group, 9 genes had more than 10 peaks, 93 genes had 6 to 10 peaks, 92 genes had 5 peaks, and the remaining genes had less than 5 peaks (Figure 5A). For ATPAF1 and FOS, which are CK and T group-specific peak-related genes, the peaks in the proximal promoter 2 kb region of the corresponding gene are presented in Figure 5B,C, respectively. The chromatin accessibility of ATPAF1 was higher in the CK group than in the T group; the opposite result was determined for the chromatin accessibility of FOS. GO and KEGG pathway enrichment analyses were conducted with the genes in the two groups (Q < 0.05). Based on the KEGG pathway results, the specific peak-related genes in the T group were enriched in Fc gamma R−mediated phagocytosis, MAPK signaling pathway, bacterial invasion of epithelial cells, regulation of actin cytoskeleton, adherent junction, tight junction, focal adhesion, endocytosis, and autophagy pathways (Figure S2A). However, the specific peak-related genes in the CK group were not significantly enriched in any of the KEGG pathways. The results suggest that *P. multocida* infection actively mobilizes the host immune response with inflammatory phenotype. Based on the GO analysis results, GO terms that were significantly enriched in the CK group were less than GO terms that were significantly enriched in the T group (Figure S2B).

### 2.5. Peak-Related MOTIF Analysis and Transcriptional Factors Enrichment

The existence of any specific known TF motifs on the top 400 enriched peaks of the two experimental groups were analyzed using MEME−AME. As a result, 51 and 49 known transcription factors were predicted in the CK and T group, respectively (Figure S1). Among them, 35 TF motifs were common to both groups, and 16 and 14 specific TF motifs were detected in the CK and T groups, respectively.

### 2.6. Corresponding Genes Analysis of the Different Peaks between the Two Groups

**Figure 6:**
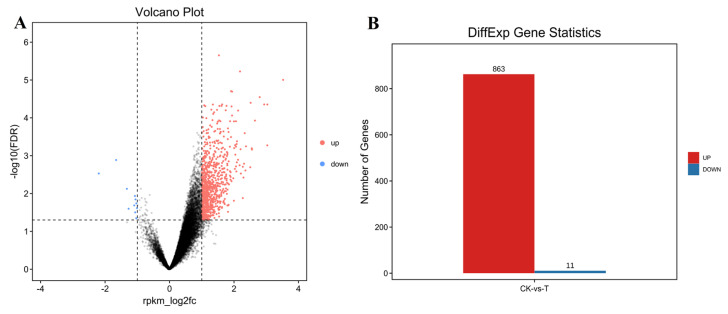

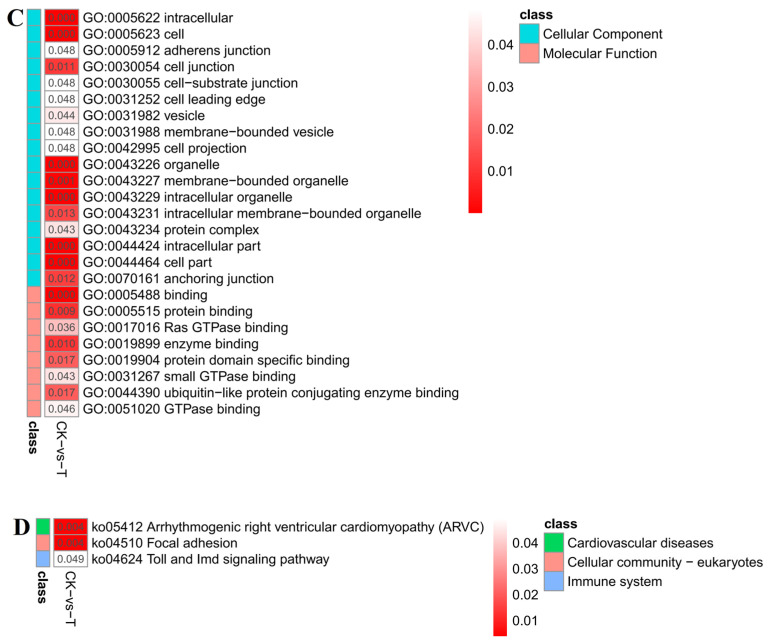
Enrichment analyses of the differential peaks and their corresponding genes. (**A**) Scatter plot of differential peaks (|log2(fold change)| ≥ 1, FDR ≤ 0.05). (**B**) Statistics for the differential peaks. (**C**) GO enrichment analysis of genes corresponding to different peaks between the two groups (Q < 0.05). (**D**) KEGG pathway enrichment analysis of genes corresponding to different peaks between the two groups (Q < 0.05).

To determine the effects of *P. multocida* infection, different peaks were filtered with FDR ≤ 0.05 and |log2(fold change)| ≥ 1 and annotated to the corresponding genes. Compared to those in the CK group, 863 and 11 peaks were upregulated and downregulated in the T group (Figure 6A,B). By annotating each peak, a total of 820 genes were obtained and used for GO analysis and KEGG pathway enrichment analysis (Q < 0.05). Cell junction and cytoskeleton organization GO terms were significantly enriched (Figure 6C). KEGG pathway enrichment analysis revealed enrichment in the Toll and Imd signaling pathway, focal adhesion, and arrhythmogenic right ventricular cardiomyopathy pathways (Figure 6D).

### 2.7. Transcription Factor Motif Analysis of Different Peaks between the Two Groups

**Figure 7:**
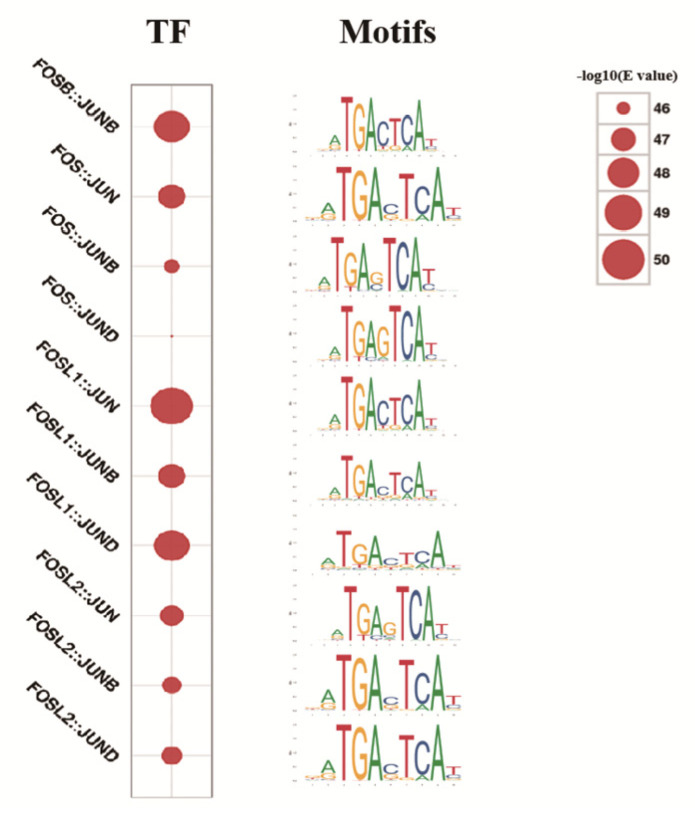
The top ten enrichment of the transcription factor motifs for different peaks between the CK and T groups. The ordinate is the TF name, and the abscissa is the comparison between two groups. The size of the circle represents the -log10 (E-value) of the corresponding TF motif.

Transcription factor motif analysis of the differential peaks between the CK and T groups was performed, and the results presented in Figure 7. Compared to the CK group, 56 known TF motifs were identified in the upregulated peaks in the treatment group (Table S1). However, no TF was identified in the downregulated peaks.

### 2.8. Differentially Expressed Genes Identified by RNA−Seq

**Figure 8:**
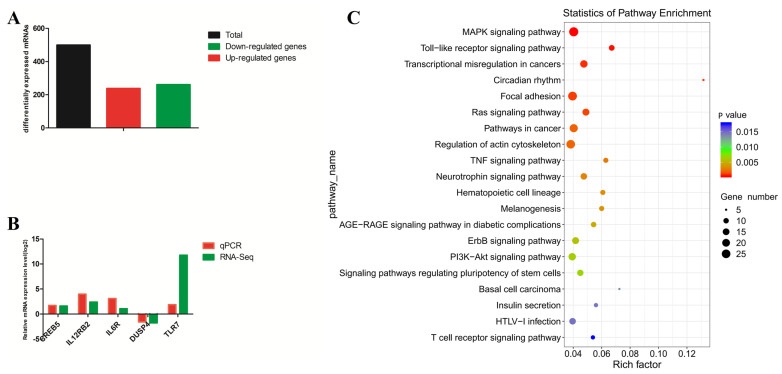
Analyses of the RNA-seq results. (**A**) Statistical numbers of differentially expressed genes (log2 FC| ≥ 1 and *p* < 0.05) in goat bronchial epithelial cells, with or without *P. multocida* infection. (**B**) The fold change trend analyses of DEGs (CREB5, IL12RB2, IL6R, DUSP4 and TLR7) obtained by qPCR and RNA−Seq. The y axis represented log2 FC for RNA−seq and log2 ^(2−△△ct)^ for qPCR data, respectively. (**C**) The top 20 significantly enriched KEGG pathways for the 499 differentially expressed genes.

To determine the effects of *P. multocida* HN01 on gene expression in goat bronchial epithelial cells, the expression of genes in the treatment group was compared with that in the CK group. Compared to those in the CK group, a total of 499 DEGs were detected in the T group, of which 238 were upregulated and 261 genes were downregulated (log2(Fold-change)| ≥ 1 and *p* < 0.05) (Figure 8A and Table S2). In order to validate the RNA−seq results, RT−qPCR was used to detect the expression levels of five differential expression immune genes (CREB5, IL12RB2, IL6R, DUSP4 and TLR7). The expression trends of genes corresponded with the results from RNA−seq (Figure 8B). KEGG pathway enrichment analysis revealed that the MAPK signaling pathway, Toll-like receptor signaling pathway, regulation of actin cytoskeleton, TNF signaling pathway, and T cell receptor signaling pathway were among the enriched pathways (Figure 8C).

### 2.9. Integrated Analysis of Specific Peak−Associated Genes and DEGs in Different Groups

**Figure 9:**
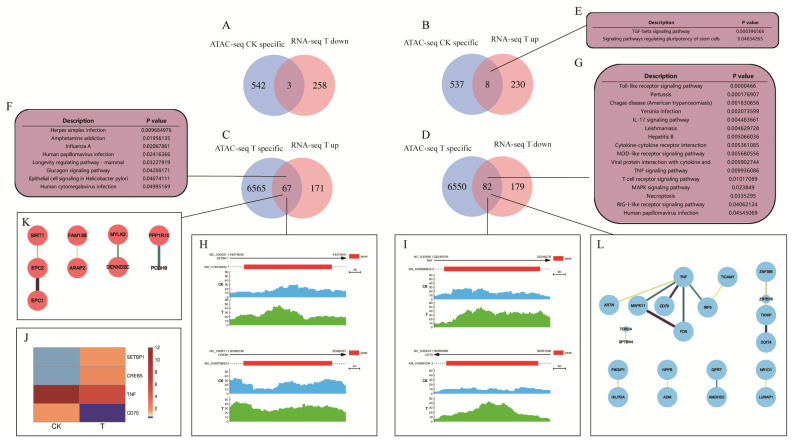
Identification of the regulatory DNA elements in the different groups. (**A**–**D**) Venn overlap of the differential peak-associated genes identified by ATAC-seq and the DEGs detected by RNA-seq. ATAC-seq T specific: T group-specific peak-related genes; ATAC-seq CK-specific: CK group-specific peak-related genes; RNA-seq T down: DEGs downregulated in the T group based on RNA-seq; RNA-seq T up: DEGs upregulated in the T group based on RNA-seq. (**E**–**G**) The significantly enriched KEGG pathways for the overlapped genes involved in the host immune response. (**H**,**I**) Peak diagram showing the genes in the chromatin open regions of the proximal promoter (2 kb) of genes (SETBP1, CREB5, TNF, and CD70). (**J**) Heatmap of the expression level of four genes (SETBP1, CREB5, TNF, and CD70) based on RNA-seq. (**K**,**L**) Protein–protein interaction network of the 67 and 82 overlapped genes. The upregulated genes are colored red, and the downregulated genes are colored green. The depth of the line represents the degree of association, and the size of the dot represents the significance of the *p*-value.

The specific peak-associated genes in the CK and T groups were used to compare the DEGs. The genes were divided into four groups for the comparison (Figure 9A–D): (1) 545 genes specific to the T group based on ATAC−seq were compared with the 261 downregulated genes in the T group based on RNA−seq. The Venn diagram results revealed 3 common overlapping genes; (2) the Venn diagram was generated by comparing 545 genes specific to the CK group based on ATAC−seq and 238 upregulated genes in the T group (downregulated in the CK group) based on RNA−seq, which revealed an overlap of 8 genes; (3) the 6632 genes specific to the T group based on ATAC−seq and 238 upregulated genes in the T group based on RNA−seq were compared, leading to an overlap of 67 genes; (4) the 6632 genes specific to the T group based on ATAC−seq were compared with the 261 downregulated genes in the T group based on RNA−seq. The Venn diagram results revealed an overlap of 82 genes. For the above overlapping parts, GO and KEGG enrichment analyses were carried out. Among the significantly enriched KEGG pathways, the host immune reaction−related pathways are displayed in Figure 9E–G.

Among the 67 genes, several genes were analyzed to determine the chromatin accessibility and RNA expression levels. SETBP1 and CREB5 had specific peaks in the T group, but no peak in the CK group (Figure 9H); the RNA expression levels of SETBP1 and CREB5 were higher in the T group than in the CK group (Figure 9J). TNF and CD70 had specific peaks in the T group, but no peak in the CK group (Figure 9I); the opposite result was obtained for the RNA expression level (Figure 9J). According to the functional annotation of the eight overlapped genes between the specific peaks associated with the CK group and the upregulated genes in the T group based on RNA-seq, two KEGG pathways and 126 GO terms were significantly enriched (*p* < 0.05) (Figure 9E). The enriched KEGG pathways were TGF−β signaling pathway and signaling pathways regulating pluripotency of stem cells. The enriched GO terms are listed in Table S3 in Supplementary Material. The function annotation results of the 67 overlapped genes revealed that eight KEGG pathways and 626 GO terms were enriched (*p* < 0.05) (Table S3). The enriched KEGG pathways included herpes simplex infection, human papillomavirus infection, epithelial cell signaling in *Helicobacter pylori* infection, and human cytomegalovirus infection (Figure 9F). Based on functional annotation of the 82 overlapped genes between the specific peaks associated with the T group and the downregulated genes in the T group by RNA-seq, 25 KEGG pathways and 426 GO terms were significantly enriched (*p* < 0.05). The host immune-related KEGG pathways are presented in Figure 9G. The enriched GO terms are listed in Table S3. There were no significantly enriched KEGG pathways and GO terms for the three overlapping genes between the specific peak−associated genes in the CK group and the downregulated genes in the T group by RNA−seq. The 67 and 82 genes obtained, respectively, from ATAC−seq and RNA−seq were further used to generate the protein−protein interaction network (PPI network) displayed in Figure 9K,L.

## 3. Discussion

Consistent efforts are being made to better understand the mechanism of *P. multocida* infection. RNA−seq was carried out to determine the RNA expression change in the lung of different animals after *P. multocida* infection. Although a change in RNA expression change was identified, the regulation mechanism was not revealed. More recently, ATAC−seq has been used to identify the accessible chromatin under different pathological conditions [13]. To highlight the regulatory elements of *P. multocida* infection in goat bronchial epithelial cells, three replicates of goat bronchial epithelial cells in the CK and T groups were collected and subjected to ATAC−seq. Samples were also used for RNA−seq, which was integrated with ATAC−seq to explore the gene expression regulation mechanism (Figure 1). Regarding the infection time and infection amount of *P. multocida*, we conducted a preliminary experiment and determined that infection for 4 h with MOI of 100 is more appropriate. We first observed morphological changes in goat epithelial cells over different time periods and different doses when detecting the degree of damage caused to the cells by invading bacteria. When infected the cells at a MOI of 100 for 4 h to 6 h, the cells became rounded and detached, some cells died, and the number of *P. multocida* increased. After 6 to 8 h of infection, almost no normal cells were observed, along with significant multiplication of *P. multocida*. Therefore, we selected a MOI of 100 and a stimulation time of 4 h as the infection conditions for subsequent ATAC−seq experiment. For the RNA−seq experiment, the cells were infected for 6 h with MOI of 100. The reason is that compared with the transcriptome data of the previous *P. multocida*−infected cells for 4 h with MOI of 100 [9], it was determined that there were almost no differentially peak-related genes gained from ATAC−seq overlapped with the differentially expressed genes gained from RNA−seq. We speculated that it may take a certain time for gene expression to be transcribed from DNA to RNA. Therefore, we specially selected cells for RNA seq infection for 6 h with the same MOI value.

A total of 13,079 and 28,722 peaks were identified for the CK and T groups, respectively (Table 2). The number of peaks in the *P. multocida*−infected group was greater than that in the CK group (Figure 4A, B). Notably, *P. multocida* infection triggers a series of immune stress responses in goat bronchial epithelial cells, leading to the opening of chromatin of multiple genes for transcriptional regulation of RNA expression levels, and finally the resistance to pathogen invasion. Compared to the CK group, the T group had more upregulated peaks (863 peaks) than downregulated peaks (11 peaks), which confirms that chromatin accessibility in the T group was greater than that in the CK group.

Based on KEGG analysis of the 6632 specific peak-associated genes in the T group, under the inflammation conditions initiated by *P. multocida* infection, actomyosin reorganization occurred, causing significant enrichment of the KEGG pathways for the regulation of actin cytoskeleton (Figure S2A,B). The macromolecule permeability of cells increases and might be regulated by the transient breakage of tight junction strands (i.e., the dynamic strand model) [14]. Notably, the tight junction was enriched in the specific peak−associated genes in the T group (Figure S2A). Thus, *P. multocida* infection impacts and damages the tight function of goat bronchial epithelial cells. The tight junction in the epithelia serves as a gate and barrier [15]. Notably, the adherens junction and focal adhesion were also enriched (Figure S2A). Tight junctions are located at the most apical region of cell−cell contacts, while adherens junction is located under the tight junction [15]. During *P. multocida* infection, both the tight junction and adherens junction are affected. Focal adhesions serve as dynamic signaling hubs within the cell that connect intracellular actin to the extracellular matrix and respond to environmental cues [16], such as host immune reaction after *P. multocida* infection.

After infection, some infectious disease pathways were significantly enriched, such as bacterial invasion of epithelial cells and Shigellosis. The regulation of actin cytoskeleton was also affected, the adherens junction and tight junction were damaged, and the host immune system was activated, incurring such action as Fc gamma R−mediated phagocytosis [17]. Additionally, endocytosis and autophagy pathways were enriched (Figure S2A). Endocytosis involves the regulation of the ways in which cells interact with their environments by controlling the lipid and protein composition of the plasma membranes [18]. Autophagy, an important part of the innate immune response and adaptive immune response, plays an important role in the defense against bacterial infection. Autophagy is also a cellular catabolic process that results in lysosome-mediated recycling of organelles and protein aggregates, and the destruction of intracellular pathogens [19]. Bacteria can evade autophagy by destroying or utilizing autophagy virulence proteins or related molecules [20]. In innate immunity, autophagy is the effector response of activation receptors, such as Toll−like receptors (TLRs) and NOD−like receptors in response to pathogens and damage-associated molecular pattern molecules. In this study, the NOD-like receptor signaling pathway was enriched by the specific peak−associated genes in the T group. In the cell, the autophagic pathway has several functions, including selective degradation of intracellular pathogens, removal of excess or damaged organelles, and elimination of potentially toxic protein aggregates. The pathway is also involved in the degradation of proteins and other macromolecules to deliver essential anabolic nutrients under conditions of nutritional stress or initiation [21].

By comparing the 51 and 49 motifs significantly enriched by unique peaks in the CK and T groups, respectively, 35 motifs were determined to overlap, and 16 and 14 motifs were different in the CK and T groups. The 35 overlapped TFs were mostly members of the activator protein 1 (AP−1) family (Figure S3). The ubiquitous family of AP−1 dimeric transcription complexes is involved in virtually all cellular and physiological functions. Cells must reprogram gene expression in response to different cues [22]. Although AP-1 has been implicated in various severe diseases as a potential overlapped TF, it is essential to the maintenance of normal cell function by regulating gene expression via transcription−pioneering−, chromatin−remodeling− and chromatin accessibility−maintenance effects [22]. Therefore, the AP−1 family of transcription factors was analyzed and observed in the T group and CK group.

After integrational analysis of the RNA−seq and ATAC−Seq results, Interferon regulatory factor 5 (IRF5) was identified as a T specific peak−associated gene; however, its transcription level was found to be lower in the T group than the CK group (Figure 9L). Although two specific peaks were identified for the IRF5 gene in the T group, they were not located 2 kb upstream of the TSS of IRF5 gene in the T group and their regulatory function was limited. IRF5 belongs to a family of transcription factors, is a central regulator of the inflammatory response and is involved in the expression of pro−inflammatory cytokine responses to microbial infection [23], most likely due to the activation of IRF5 via the toll-like receptor (TLR) −MyD88 pathway [24]. In this pathway, TLR activation by TLR ligands, including pathogen LPS, R848, and CpG, induces a signaling cascade [25,26]. However, TLR2 and TLR6 are T group specific peak-associated genes, and the Toll and Imd signaling pathways were enriched between the two groups.

TNF proinflammatory cytokine and CD70 belong to the tumor necrosis factor (TNF) superfamily. CD70 interacts with its unique receptor, CD27, which is bound to TNF receptor-associated factors (TRAFs), such as TRAF2 and TRAF5, thereby activating the nuclear factor-kappa B (NF-κB) and c-Jun kinase pathways, enabling activation of innate and adaptive immunity, which is crucial for the regulation of the cellular immune response [27,28,29]. The overexpression of CD70 can be observed in different auto-immune diseases, such as rheumatoid and psoriatic arthritis [27,30,31]. However, in this study, specific peaks of CD70 were observed in the T group (Figure 9I). Specifically, the expression level of CD70 was decreased in the T group (Figure 9J). Hence, some potential transcriptional repressors might be involved in the regulation of CD70 gene expression in host immune reaction to *P. multocida* infection. TNF has been identified as a key regulator of the inflammatory response in infectious diseases [32,33]. TNF can bind to its receptors, TNFR1 and TNFR2, which initiate pro-inflammatory signals through NF-κB activation. NF-κB is a rapidly acting primary transcription factor present in all cell types and is involved in the cellular responses to stimuli, such as cytokines and stress. NF-κB also plays a key role in regulating the immunological response to infections [34]. In the current study, the specific peaks of TNF were detected in the T group (Figure 9I), which may enable the binding of TNF to its receptor and the initiation of signal transduction pathways that include NF−κB to induce a cellular response to *P. multocida* infection.

Sixty-seven genes with specific peaks in the T group and a higher transcription level than that in the CK group were obtained (Figure 9C). Among them, SETBP1 had eight peaks and was upregulated after *P. multocida* infection by binding to the SET nuclear oncogene, which plays an important role in the process of DNA replication (Figure 9H,J). The overexpression of SETBP1 promotes the proliferation of leukemia and is closely associated with poor overall survival (OS) in patients with AML, specifically elderly patients [35]. Thus, the immune response to *P. multocida* invasion of bronchial epithelial cells occurs via the regulation of SETBP1 based on the peaks and mRNA expression. Following *P. multocida* infection, the RASGEF1B peaks and RNA expression were upregulated in goat bronchial epithelial cells; this is because RasGEF1B plays a functional role in the process of cellular response to LPS stimulation of *P. multocida* [36]. The cell immune reaction occurs via the Toll−like Receptor (TLR) and is regulated by NF-κB through its proximal promoter [37]. cAMP responsive element binding protein 5 (CREB5) was upregulated and had four specific peaks in the T group. CREB5 is a transcriptional activator in eukaryotic cells and belongs to the ATF/CREB family. CREB5 can regulate gene expression by specifically binding to CRE as a homodimer or c−Jun or CREBP1 as a heterodimer. Notably, CREB5 functions as a CRE−dependent trans−activator [38] and its presence activates large amounts of gene expression, leading to more specific peaks in the T group for the response to *P. multocida* infection.

Based on the results of current study, it is suggested that some potential transcription factors and genes play crucial roles in the host’s response to *P. multocida* infection, such as SETBP1, RASGEF1B, CREB5, IRF5, TNF, and CD70. In the subsequent study, these genes will be further studied to explore their function and mechanism during the host’s resistance to *P. multocida* infection so as to provide clues for breeding resistant goat populations. In conclusion, to explore the mechanism involved in the changes in cell morphology and transcriptome, ATAC−seq was performed and the changes in the chromatin open region of *P. multocida*-infected goat bronchial epithelial cells were assessed based on transcriptional regulation. The accessible regions of host chromatin were determined to significantly increase after *P. multocida* infection. Furthermore, the annotated genes in these regions were significantly enriched in the pathways related to immune response, such as cell infection, endocytosis, and adhesion junction. Differential peaks were most significantly enriched in the MAPK signal and tryptophan pathways. Overall, the findings serve as valuable information for understanding the regulatory mechanisms of *P. multocida*−infected goat bronchial epithelial cells and establishing goat population genetic improvement programs.

## 4. Materials and Methods

### 4.1. Bacterial Preparation

The *P. multocida* strain HN01 was isolated from the lung of a Hainan black goat and identified as serotype D (GenBank accession No. cp037861.1) by our lab previously, and was used for the infection experiment. The bacteria were revived from −80 °C and cultured overnight in a tryptic soy broth (Hopebio, Qingdao, China) supplemented with 5% (*v*/*v*) bovine serum (HyClone, Logan, UT, USA) at 37 °C for 12 h using an orbital shaker before use in the infection assays. The concentrations for the infection assays were determined by plating serial dilutions on plate count agar, and 2.3 × 10^9^ CFUs (colony−forming units) of *P. multocida* HN01 in 1 mL of cell culture medium were used for the infection.

### 4.2. Cell Culture and Infection

Goat bronchial epithelial cells were isolated from goat and cultured in PriMed−iCell−001 medium supplemented with 2% fetal bovine serum (FBS) (iCell Bioscience Inc, Shanghai, China). Briefly, goat bronchial was isolated, sterilized, and minced to approximately 1 cm^3^ size pieces in the cell culture plates with 2 mL medium, were inverted and cultured at 37 °C in a 95% humidified atmosphere with 5% CO_2_ for 2 h. The cell culture plate was then immediately inverted to upright and the cells further cultured until confluence occurred into pieces. The bronchial tissue block was then removed, and the remaining adherent cells were digested with 5% trypsin (HyClone, Logan, USA) for the passage. For the infection assays, the cells were seeded in six−well plates with an average of 4.5 × 10^5^ cells in each as counted with a hemocytometer. The cells were incubated at 37 °C in a 95% humidified atmosphere with 5% CO_2_ until more than 80% of confluence of cells occurred. The experiment was assigned into two treatments, including the control (CK) group and *P. multocida* HN01 infected (T) group, cells in the CK group were administered 1 mL of cell culture medium, whereas the goat bronchial epithelial cells in T group were incubated with 1 mL of the infection suspensions (2.3 × 10^9^ CFUs of *P. multocida* HN01 with MOI of 100). After 4 or 6 h of infection, the infection suspensions were removed and the cells were washed 3 times with cell culture medium containing 10% FBS and 200 μg/mL gentamycin to remove extracellular bacteria. Cells were then washed once with PBS. Three replicates of the 4 h treatment were digested with 5% trypsin and collected for nuclear extraction, and another three replicates treated for 6 h were used for RNA extraction (Figure 1).

### 4.3. ATAC−Seq and Bioinformatics Analysis

The nucleus of cells in three replicates in CK and T groups were lysed by pyrolysis liquid with 10 mM Tris−HCl (pH 7.4), 10 mM NaCl, 3 mM MgCl_2_, and 0.1% (v/v) Igepal CA−630 (Beyotime, Shanghai, China). After centrifuging for 5 min under 500 rpm at 4 °C, the nuclei were obtained. Transposition reaction was performed using TruePrep^®^ DNA Library Prep Kit (Vazyme, Beijing, China) at 37 °C for 30 min containing Tn5 transposase. The libraries were amplified for 15 cycles of 98 °C for 15 s, 60 °C for 30 s and 72 °C for 3 min using TruePrep^®^ DNA Library Prep Kit (Vazyme). The products were purified using the QIAGEN MinElute PCR purification kit (QIAGEN, Dusseldorf, Germany). The libraries were sequenced using the Illumina HiSeq^TM^ 4000 platform at Gene Denovo Biotechnology Co. (Guangzhou, China). Raw reads from sequencing were processed to obtain high−quality clean reads by filtering the adapters and low-quality bases according to three stringent filtering standards: (1) removal of reads containing adapters, (2) removal of reads containing more than 10% unknown nucleotides (N), and (3) removal of low-quality reads containing more than 50% of low-quality (Q-value ≤ 20) bases. Filtered reads of each sample were mapped to the mitochondrial genome (NC_005044.2) in reference genome (GCF_001704415.2) using Bowtie2 (version 2.2.8) with the parameters–X2000 [39]. Reads aligned to the mitochondrial genome were removed and remaining reads that were aligned specifically in the genome (GCF_001704415.2) were finally used for subsequent analysis. All reads aligned to the sense strand were offset by + 4 bps, and all reads aligned to the anti-sense strand were offset by −5 bps. After shifting, the aligned paired reads were used for peak calling on MACS (version 2.1.2) [40], using parameters “--nomodel --shift -100 --extsize 200 -B -q 0.05”. Dynamic Poisson distribution was used to calculate the *p*-value of the specific region based on the unique mapped reads. *p*−value was then corrected by false discovery rate (FDR) to obtain the Q−value. Dynamic Poisson distribution was used to calculate the *p*-value of the specific region based on the unique mapped reads. The region is defined as a peak when the final Q−value is less than 0.05. According to the genomic location information and gene annotation information of the peak, peak−related genes were confirmed using ChIPseeker (version v1.16.1) [41].

### 4.4. Differential Analysis of Multi-Samples

DiffBind (version 2.8.0) was used to analyze peak differences across groups [42]. Significant differential peaks were identified with FDR ≤ 0.05 and |log2 FC| ≥ 1 in the two comparison groups. Peaks that located less than 2 kb of the transcription start site (TSS) and no more than 500 bp in the upstream of TSS were analyzed using functional enrichment analysis. The Cluster Profile package in R was used to analyze the Gene Ontology (GO)/Kyoto Encyclopedia of Genes and Genomes (KEGG) enrichment pathway of the differential peaks−associated genes.

### 4.5. Motif analysis

The DNA binding site for specific transcription factors (TF) always display a conserved DNA sequence pattern. MEME-AME in MEME suit (http://meme-suite.org/ (accessed on 30 January 2022)) was used to detect and confirm the existence of any known TF-motifs. For the significance motif in the top 400 enriched peaks, each peak sequence was scanned with a motif that has been reported for the existing transcription factors. The significance of the enriched transcription factor motif was determined using Fisher’s exact test.

### 4.6. RNA−Seq and qRT−PCR Validation

RNA−seq of goat bronchial epithelial cells was performed under the same conditions used for ATAC−seq. Total RNA was extracted by Trizol (Transgen, Beijing, China) according to the manufacturer’s procedure. After the quality detection, the RNA was reverse transcribed and used for cDNA library construction. Sequencing and data analysis were performed as described elsewhere [9,10]. Briefly, the transcriptome data were assembled using StringTie and compared with the reference genome using Hisat. StringTie and edgeR were used to estimate the expression levels of all transcripts. The calculated gene expression was used directly to compare gene expression between different samples. Significantly differentially expressed genes (DEGs) were obtained by screening with |log2(fold change)| ≥ 1 and *p*-value < 0.05 criterion in the R package. Three biological replicates were used. Quantitative RT−PCR was used to validate the expression of five differentially expressed mRNAs (CREB5, IL12RB2, IL6R, DUSP4, TLR7, and GAPDH). Total RNA was converted to first strand cDNA by using EasyScript^®^ Reverse Transcriptase (Transgen) and qPCR was performed using a TransStart^®^ Green qPCR SuperMix (Transgen) with the following PCR cycling conditions: an initial denaturation step at 95 °C for 30 s, 40 cycles at 95 °C for 10 s, annealing temperature for 30 s and 72 °C for 10 s. The PCR primers were designed and listed in Table 3. The relative expression of the target genes was calculated by using the 2^−△△Ct^ method and normalized to the expression of GAPDH. The results were expressed as fold change relative to the average value of the CK group. All the experiments were conducted independently three times.

### 4.7. Integrated Analysis of ATAC−Seq and RNA−Seq

To explore the relationship between open chromatin accessibility and gene transcription levels, the genes associated with the open chromatin regions in the two experiment groups were overlapped with the upregulated and downregulated genes in the same experiment transcriptomes, respectively. The target genes were mapped to the protein in the database to search for homologous protein. The interaction network was built according to the interaction relationship of the homologous protein using String software. The completed protein interaction network was transferred to Cytoscape (version 3.7.2) for visualization.

## Figures and Tables

**Table 1:** ATAC-seq data statistics.

Sample	Raw Reads	Clean Reads (%)	Mitochondria Mapped Reads (%)	Goat Mapped Reads (%)	Pasteurella Mapped Reads (%)
CK-1	43,714,258	42,997,192 (98.36%)	3,504,534 (8.02%)	39,163,518 (89.59%)	4862 (0.01%)
CK-2	28,669,772	27,967,118 (97.55%)	2,324,160 (8.11%)	24,433,370 (85.22%)	3548 (0.01%)
CK-3	28,947,450	28,242,082 (97.56%)	2,603,258 (8.99%)	24,526,906 (84.73%)	4744 (0.02%)
T-1	48,363,990	47,514,406 (98.24%)	3,217,946 (6.65%)	43,472,734 (89.89%)	6566 (0.01%)
T-2	41,106,300	40,322,350 (98.09%)	2,538,132 (6.17%)	36,377,884 (88.5%)	6618 (0.02%)
T-3	42,871,856	42,062,878 (98.11%)	3,014,546 (7.03%)	37,990,568 (88.61%)	6436 (0.02%)

**Table 2:** Statistical table for the peak of each sample.

Sample Id	Peak Number	Total Length	Average Length	Genome Ratio
T-1	33,333	11,792,578	353	0.40%
T-2	39,685	15,043,263	379	0.51%
T-3	26,048	9,125,900	350	0.31%
T-common	28,722	14,398,456	501	0.49%
CK-1	16,622	5,357,070	322	0.18%
CK-2	12,829	3,920,020	305	0.13%
CK-3	16,500	5,291,746	320	0.18%
CK-common	13,079	5,496,690	420	0.19%

**Table 3:** Primers used for qPCR analyses.

Gene Names	Primer Sequence (5′-3′)	Product Size (bp)
CREB5	F: AGCAGAACCACCCACATC R: ATCCTCATCCACCACCCT	197
IL12RB2	F: TGTTCACTGGCACTTACTT R: GCCTTGTTTGGGCTTCA	153
IL6R	F: GGCAACATCTCAGTCAGCG R: CCACTCCAGGCATCACG	197
DUSP4	F: AGCACAGCGGAGTCTTTGGA R: CGAAGTGGTTTGGGCAGTCA	190
TLR7	F: CACGCCCATCTTTGACTTCG R: CACCAGGACCAGGCTCTTCT	151
GAPDH	F: CTCTCTGCTCCTGCCCGTTC R: TGTGCCGTGGAACTTGCCAT	241

F and R represented the forward and reverse primers, respectively.

## Data Availability

The raw data of RNA-seq and ATAC-seq are available in the BIG Data Center (http://bigd.big.ac.cn/) under the Bioproject PRJCA010994 and PRJCA012902, respectively.

## Supplementary Materials

The following supporting information can be downloaded at: https://www.mdpi.com/article/10.3390/ijms24021312/s1.
