# Supplementary material for: Profiling Chromatin Accessibility Responses in Goat Bronchial Epithelial Cells Infected with Pasteurella multocida
Source: Int J Mol Sci. 2023 Jan 9;24(2):1312. doi: 10.3390/ijms24021312 (PMC9861026; doi:10.3390/ijms24021312)
Supplement: Supplementary file 1 [file ijms-24-01312-s001.zip › Figures S1-S3.docx]

**Figure S1.** Principal component analysis (PCA) of the samples. Different colors represented different groups, each with three replicates.

(**A**)

(**B**)

**Figure S2.** Functional analysis of specific peak-related genes in the CK and T groups. (A) KEGG pathway enrichment analysis of the specific peak-related genes in the different groups (Q < 0.05). (B) GO enrichment analysis of the specific peak-related genes in the different groups (Q < 0.05). CK-vs-T.CK and CK-vs-T.T represented that specific peak-related genes in the CK and T group, respectively. CK-vs-T.common represented that the common peaks-related genes between CK and T group. The number on each rectangle represented the Q value of each treatment genes enrichment in the corresponding KEGG pathway or GO term.

**Figure S3.** Enrichment of the transcription factor motifs for specific peaks in the CK and T groups. The ordinate is the TF name, and the abscissa is the comparison between two groups. The size of the circle represents the E-value of the corresponding TF motif.
